# Combining Surgical Innovations in Amputation Surgery—Robotic Harvest of the Rectus Abdominis Muscle, Transplantation and Targeted Muscle Reinnervation Improves Myocontrol Capability and Pain in a Transradial Amputee

**DOI:** 10.3390/medicina59122134

**Published:** 2023-12-07

**Authors:** Jennifer Ernst, Janne M. Hahne, Marko Markovic, Arndt F. Schilling, Lisa Lorbeer, Marian Grade, Gunther Felmerer

**Affiliations:** 1 Department of Trauma Surgery, Orthopedic Surgery and Plastic Surgery, University Medical Center Göttingen, 37075 Göttingen, Germany; uni@janne.de (J.M.H.); marko.markovic@med.uni-goettingen.de (M.M.); arndt.schilling@med.uni-goettingen.de (A.F.S.); gunther.felmerer@med.uni-goettingen.de (G.F.); 2 Department of Trauma Surgery, Hannover Medical School, 30625 Hanover, Germany; lorbeer.lisa@mh-hannover.de; 3 Department of General, Visceral and Pediatric Surgery, University Medical Center Göttingen, 37075 Göttingen, Germany; marian.grade@med.uni-goettingen.de

**Keywords:** robotic surgery, targeted muscle reinnervation, transplantation, myoelectric prostheses, pattern recognition, nerve transfer

## Abstract

Adding robotic surgery to bionic reconstruction might open a new dimension. The objective was to evaluate if a robotically harvested rectus abdominis (RA) transplant is a feasible procedure to improve soft-tissue coverage at the residual limb (RL) and serve as a recipient for up to three nerves due to its unique architecture and to allow the generation of additional signals for advanced myoelectric prosthesis control. A transradial amputee with insufficient soft-tissue coverage and painful neuromas underwent the interventions and was observed for 18 months. RA muscle was harvested using robotic-assisted surgery and transplanted to the RL, followed by end-to-end neurroraphy to the recipient nerves of the three muscle segments to reanimate radial, median, and ulnar nerve function. The transplanted muscle healed with partial necrosis of the skin mesh graft. Twelve months later, reliable, and spatially well-defined Hoffmann–Tinel signs were detectable at three segments of the RA muscle flap. No donor-site morbidities were present, and EMG activity could be detected in all three muscle segments. The linear discriminant analysis (LDA) classifier could reliably distinguish three classes within 1% error tolerance using only the three electrodes on the muscle transplant and up to five classes outside the muscle transplant. The combination of these surgical procedure advances with emerging (myo-)control technologies can easily be extended to different amputation levels to reduce RL complications and augment control sites with a limited surface area, thus facilitating the usability of advanced myoelectric prostheses.

## 1. Introduction

### 1.1. Prosthetic Hand Replacement and Control

The hand is a remarkable result of evolution, providing humankind with dexterous manipulation and robust grasping. More than 27 degrees of freedom and a high sensory resolution of the fingertips allow for a unique motor function and sense of touch [1,2]. Thus, losing a hand can dramatically impact quality of life. Electronic prostheses can help here. They are typically controlled by surface electromyographic (EMG) signals generated by the remaining muscles at the residual limb (RL). The most common control approach is based on two electrodes placed in the prosthetic socket above two antagonist muscle groups, e.g., the wrist extensors and flexors in transradial amputations [3]. This enables a proportional control of a single degree of freedom (DOF); e.g., one electrode is used for opening and one for closing the hand. A switching function allows controlling a second degree of freedom, such as rotation. This sequential control strategy is slow and unnatural and is one of the reasons for the high rejection rates of prosthetic hands [4,5].

Extending direct control is not possible for most users as there are typically not enough independent control sites available at the RL. Targeted muscle reinnervation (TMR) is a surgical approach developed to mitigate this limitation [6]. Using this technique, the nerve endings at the RL are linked to the remaining muscles, which become innervated and serve as biological amplifiers. The newly innervated muscles can be controlled voluntarily and intuitively, generating additional EMG hotspots that can be used for direct proportional and simultaneous control of up to three DOFs [6,7,8,9]. Furthermore, recent studies have demonstrated that TMR reduces phantom and residual limb pain (RLP) [10,11]. This outcome is relevant since up to 80% of hand amputees suffer from amputation-related pain [12].

RLP is predominantly driven by painful neuromas and insufficient soft-tissue coverage. Both substantially impact quality of life, may prohibit prosthetic use, and may lead to additional revision surgeries.

In the TMR procedure on the transradial level, the median nerve is usually transferred to the flexor digitorum superficialis (FDS) muscle and the ulnar nerve is transferred to the flexor carpi ulnaris (FCU) muscle. However, tissue trauma, insufficient soft-tissue coverage, or short residual limbs sometimes preclude using the FCU and FDS, limiting TMR’s applicability in these patients [13,14,15].

### 1.2. The Rectus Abdominis (RA) Muscle in Reconstructive Surgery

In plastic and reconstructive surgery, the rectus abdominis (RA) muscle flap is frequently used for a multitude of indications in the coverage of soft tissue defects [16,17,18]. The RA has an axial, type III blood supply and receives a segmental motor and sensory nerve supply by the ventral rami of the thoracic nerves T7-12 entering the muscle at the aponeuroses blend on the posterolateral surface of the flap [19]. The RA muscle is divided by three horizontal fibrous bands (tendinous intersections) attributable to the original segmentation of the myotomes. An animal model using a pedicled RA demonstrated that TMR could generate a separate EMG signal in each muscle segment [20].

Conventionally, the free rectus muscle is harvested through wide incisions in the abdomen. The instability of the abdominal wall is a common complication of this procedure because it harms the anterior rectus sheath [21]. Intraperitoneal minimally invasive harvest of the rectus muscle flap through the posterior sheath has been shown to avoid and reduce the frequency of these complications [22,23].

In this case study, we integrated the concepts of robotic-assisted RA transplantation surgery with TMR for advanced prosthetic control in a transradial amputee who needed revision surgery.

## 2. Materials and Methods

### 2.1. Case

A forty-year-old male patient lost his right dominant hand in a work-related accident. Four years after the accident, he was referred to our clinic due to insufficient soft-tissue coverage and resulting unstable scars at the transradial RL. Additionally, he suffered from strong RLP, presumably caused by the neuroma. Pain levels at nine of ten on the Numeric Rating Scale (NRS) prohibited him from wearing the standard two-channel myoelectric-controlled prosthesis.

Initial clinical examination showed a free range of motion of the elbow joint. The RL length was 16 cm; however, the distal 9 cm were covered with a skin mesh graft causing unstable scar conditions and exposing the bony edges of the radius and ulna. Neuromas of the radial, ulnar, and median nerves were identified by palpation of the RL. Preoperative angiography revealed unimpaired recipient vessels allowing a free muscle flap transfer.

### 2.2. Surgical Technique

First, the recipient site was prepared: the skin mesh graft was resected at the distal RL, and the ulnar, median, superficial, and deep branches of the radial nerve were identified using loupe magnification. The recipient vessels (the brachial artery and its accompanying vein) were then prepared at the supinator muscle’s entry point.

**Figure 1:**
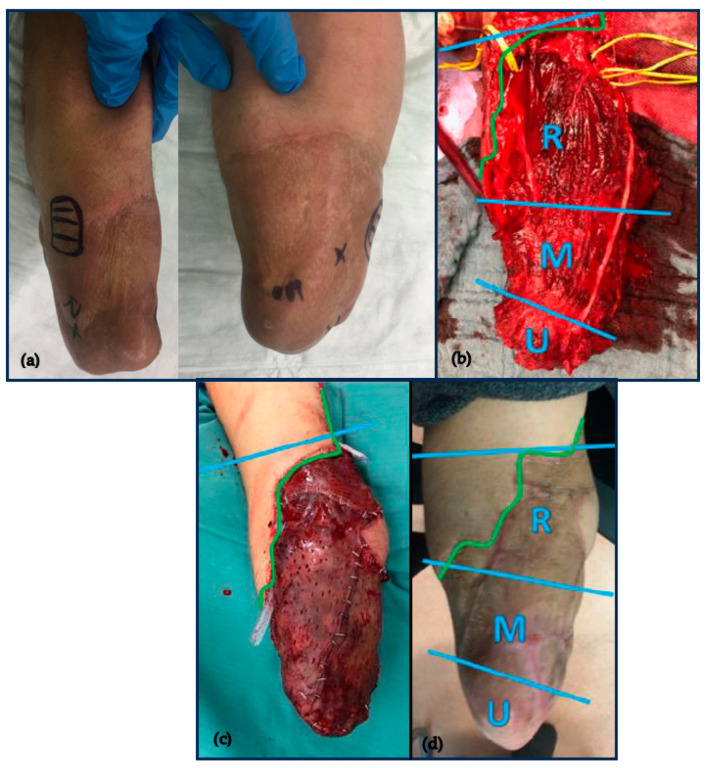
Evolution of the transradial residual limb (**a**–**d**). The proximal blue line indicates the elbow fold; the distal two blue lines the segments of the RA muscle. The green line marks the border of the RA muscle. (**a**) Preoperative pictures: the markings indicate the position of the electrodes of the previous two-channel myoelectric-controlled prosthesis and the maximum felt tingling after percussion (HT sign). (**b**) Intraoperative picture with draped nerves to indicate the dorsally to the muscle graft performed epimysial coaptation site of the radial, median, and ulnar nerves to the three segments. (**c**) The free muscle flap was covered with skin mesh graft after anastomosis and nerve transfer. (**d**) RL after wound healing eight months post-surgery.

The intraperitoneal flap was harvested using the DaVinci Xi system (Intuitive Surgical, Sunnyvale, CA, USA). Four ports were positioned at the right flank of the abdomen. Next, the left RA muscle was robotically harvested and retrieved through an incision approximately 2 cm proximal to the symphysis at the left anterior sheath exposing the flap (for detailed robotic harvesting technique of the rectus muscle, please see [22,23,24]). The harvested RA flap was then transplanted to the prepared RL. End-to–site microsurgical anastomosis of the inferior epigastric artery to the brachial artery and end-to-end anastomosis of the accompanying vein secured perfusion of the free flap. After neuroma excision, epineural-to-epimysial nerve-muscle transfer was performed. Intraoperative electrical nerve stimulation allowed the identification of the superficial and the deep branches of the radial nerve. Observing from proximal to distal, the radial, median, and ulnar nerves were transferred to the posterior sheath of the three portions of the RA muscle (Figure 1). To bridge the distance of the radial nerve stump to the proximal segment of the RA muscle, a nerve graft from the upper arm (medial cutaneous brachial nerve) was required. Finally, a partial thickness skin graft was harvested from the thigh to cover the thick muscle flap.

### 2.3. Rehabilitation Protocol

After wound healing, the patient started with physiotherapy. An occupational therapist instructed him to attempt to move the phantom hand and wrist according to a daily protocol. This approach was supported with illustrations of different hand postures. At the follow-up visits, the patient was asked if he noticed twitches related to specific phantom movement patterns.

Transcutaneous electrical stimulation was applied for ten minutes per day to prevent muscle atrophy of the RA muscle flap during the reinnervation period. Additionally, a compression socket was fitted to the RL to shape the RL and prevent scarring of the skin mesh graft.

### 2.4. Myoelectric and Sensory Assessment

**Figure 2:**
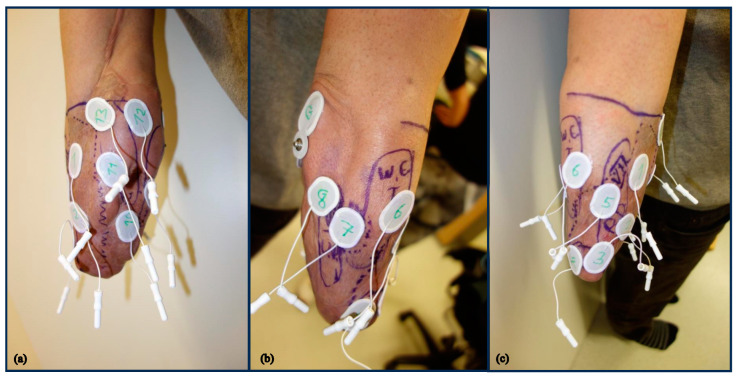
EMG recording sites, specified by palpation. Channel 1–3 on the three compartments of the transplant and channels 4–13 on neighboring muscles. (**a**) Ventromedial RL, (**b**) dorsolateral RL, (**c**) ventrolateral RL.

The EMG control capabilities of the transplant were examined using three monopolar Ag/AgCl Electrodes (Ambu Neuroline 720) placed on the skin surface at locations corresponding to the three segments of the free muscle flap, innervated by the radial, median and ulnar nerves (Figure 2). The target regions were identified by intra- and post-operative pictures revealing the location of the rectus segments and confirmed by Hoffmann–Tinel signs during moderate percussion of the RL [25].

Nine additional EMG electrodes were placed on muscles at the residual forearm that were identified by palpation during the execution of various phantom limb motions (Figure 2).

EMG was acquired using a USB-II biosignal amplifier (OT Bioelectronica, Torino, Italy) operating at 2048 Hz sampling frequency. The data were streamed to a software framework for real-time visualization developed in MATLAB (Mathworks, Natick, MA, USA). Real-time feedback on the EMG activity was depicted using a polar plot representation, which allowed for an intuitive visual inspection of the contraction patterns.

The subject was asked to conduct 13 different phantom limb motions (wrist extension, wrist flexion, pronation, supination, ulnar deviation, claw fingers, fist, thumb abduction, thumb opposition, index finger extension, index finger flexion, pinky extension, and pinky flexion). First, the subject observed the polar plot of his EMG activity and performed a few introductory trials to familiarize himself with each motion. Then, three trials of three seconds duration each were recorded with a strong but comfortable contraction level for each motion class.

### 2.5. EMG Analysis

The data were filtered with a high-pass filter (Butterworth, fourth order, Fc = 20 Hz) to remove motion artifacts, a low-pass filter (Butterworth, fourth order, Fc = 500 Hz) to remove high-frequency noise above the EMG spectrum, and a comb filter (50 Hz) was applied to remove power-line interferences, including harmonics.

First, the EMG activity of the three electrodes representing the radial-, median- and ulnar-reinnervated segments of the free muscle flap during selected phantom limb motions related to the three transplanted nerves were examined.

The applicability of the RA muscle for myoelectric control was then examined by analyzing the classification accuracy of the features of filtered EMG signals. The features were calculated on 200 ms long time windows using a 40 ms increment as the mean absolute values (MAVs) of the EMG amplitude for each time block and channel. The feature extraction parameters were selected to frame the results in the context of real-time prosthesis control, where the delay should be kept at a minimum [26]. To reduce the influence of the contraction level that was not controlled during the recordings, the features of each block were normalized by the average MAV across all channels (or all included channels in case a subset was used). Three-fold cross-validation was applied to determine classification performance: The features of all windows from two out of the three trials were used to train a multi-class linear discriminant analysis (LDA) classifier, and the remaining trial was utilized for testing. This process was repeated three times, with each trial used once for testing. The average classification error across the three folds was computed as a performance metric.

**Figure 3:**
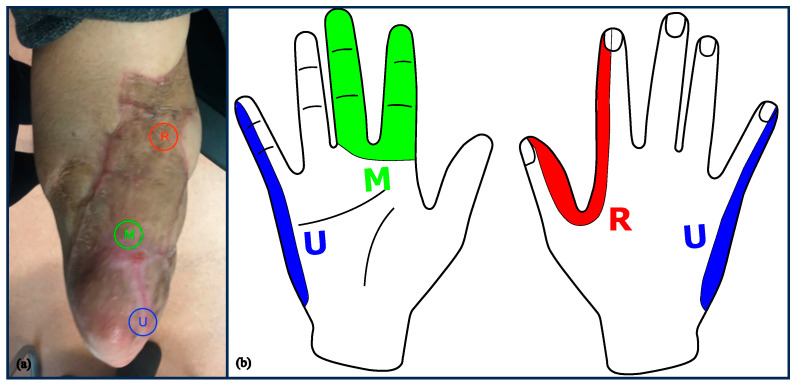
Sensation in the area of the transplant. (**a**) Locations of Hoffmann–Tinel signs at the residual limb (RL). (**b**) Phantom sensations are elicited reliably by percussion of the RL referring to ulnar, median, and radial distribution areas.

Three different electrode sets were used for training/testing the classifier: (1) only the electrodes lying on the muscle transplant (electrodes 1–3, Figure 2), (2) only the electrodes lying outside the muscle transplant (electrodes 4–13, Figure 2), and (3) all 13 electrodes.

A backward selection was used for each electrode set to select a subset of motion classes. Therefore, based on the class-specific classification error, the class with the largest error was excluded, and this process was repeated until only three classes remained in the selection.

## 3. Results

### 3.1. Clinical Course

#### 3.1.1. Wound Healing

Wound healing took three months post-surgery due to partial necrosis of the skin mesh graft. Twelve months after surgery, there were three reliable and spatially well-defined Hoffmann–Tinel signs detectable at three segments of the RA muscle flap, and 18 months after surgery, the patient did not show any donor-site morbidities. The patient could reliably assign the cutaneous stimulation by percussion to the original distal distribution areas of the ulnar, median, and radial nerve at the hand and wrist (Figure 3).

#### 3.1.2. Residual Limb Pain (RLP)

The residual limb pain was reduced from nine to six (out of ten points in NRS). This was probably because the intervention improved coverage with the muscle flap and redirected the neuromas to the muscle segments. Therefore, in contrast to the state before the intervention, the patient could now tolerate wearing the myoelectric prosthesis.

### 3.2. EMG-Activity Patterns of the Transplant

**Figure 4:**
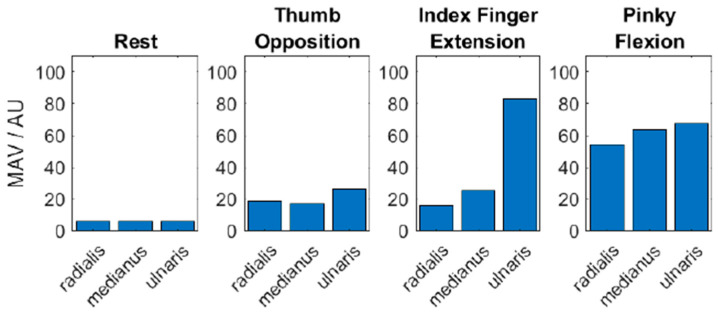
EMG activity patterns of the three segments of the RA muscle during selected phantom limb motions. All three compartments showed EMG activity clearly above baseline noise (indicated as rest), and their activation rations changed during the execution of different phantom limb motions. Interestingly, the index finger extension provides the strongest signal within the ulnar nerve segment and not on the radial segment. This can be caused by the natural co-activity of the agonist and antagonist muscles or due to the imagined motion including and hyperextension of the 5th interphalangeal joints by the lumbrical muscle. The 3rd and 4th lumbricals are ulnar-innervated intrinsic muscles of the hand that flex the metacarpophalangeal joints and extend the interphalangeal joints.

Twelve months post-surgery, EMG activity could be detected in all three compartments of the transplant. The subject could not generate fully isolated contraction patterns in each of the compartments. Instead, the three sections contracted synergistically. However, the subject could alter the activation rations between the three sections while performing different phantom limb motions (Figure 4).

**Figure 5:**
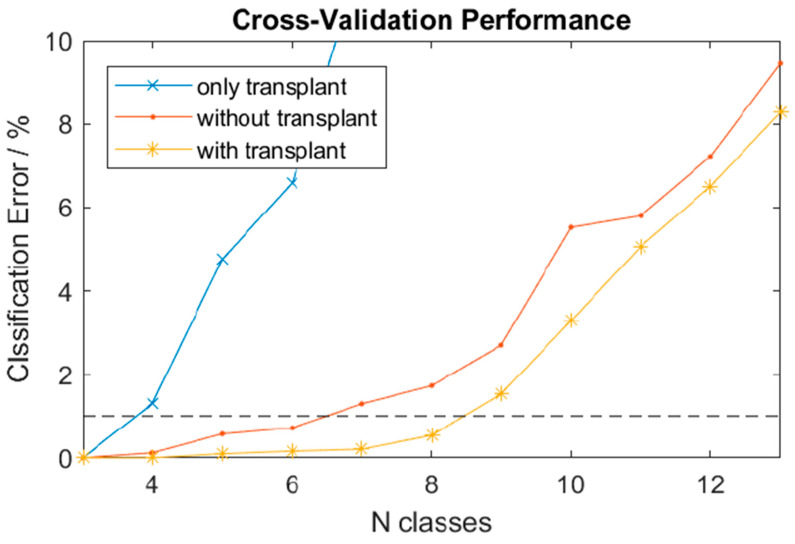
Classification accuracy using the transplant, other EMG sites, and a combination of both for a different number of classes.

The LDA classifier could distinguish several motion patterns with low classification error (Figure 5). As it is common for myocontrol applications, the classification error increased with an increasing number of classes for all electrode combinations. The threshold for the maximal tolerable error was set to 1%. Three classes could be distinguished within this error tolerance using only the three electrodes on the muscle transplant (Figure 5). Up to five classes could be detected when using the electrodes outside the muscle transplant. Finally, when all electrodes were used, the number of distinguishable classes increased to seven, i.e., two more classes could be separated by adding the transplant.

## 4. Discussion

Before the operation, the patient could not wear a prosthesis at all due to painful neuromas and insufficient soft-tissue coverage. The surgery substantially improved both problems, allowing for a prosthesis fitting, and no signs of new neuroma formations were observed. This positive outcome after revision surgery caused the health insurance to finance a prosthetic supply with a myoelectric fitting and commercially available pattern recognition system.

With TMR, it is possible to create additional myoelectric control sites, which increase the myocontrol bandwidth. However, the TMR procedure is usually confined to larger muscles with well-separated muscle compartments (such as pectoralis major). It is, therefore, less suited for transradial amputees, which leaves a significant patient group (33–45% of major upper limb amputations [27,28]) without the possibility of utilizing the benefits of this approach.

Starting from the insight that a rewiring of the residual nerves is successful in even smaller muscle recipients not exceeding some centimeters [29], we decided to harvest and transplant the RA muscle as each RA offers three separated muscle compartments. The architecture of the muscle allowed for reinnervation by selective nerve transfer.

In the past, the RA muscle had been commonly used for free muscle coverage in reconstructive plastic surgery [23]. However, the disadvantage was the invasiveness of traditional harvesting and the violation of the abdominal wall, but we circumvented this risk using robotic harvesting [23].

The Hoffmann–Tinel-sign served as a reliable, non-invasive monitoring tool during the reinnervation process [25]. The tingling sensation indicated ongoing reinnervation of the transferred arm nerves before contractions of the corresponding muscle segments became visible. At the time of the measurements, it was detected for the radialis, the median, and the ulnar nerve at the anatomically expected positions above the target segments of the free muscle flap (Figure 3). This corroborates the correct mapping for all three nerves and even the successful extension of the radial nerve to the distal segment of the transplanted free muscle with the nerve graft to reach the target area.

Although the subject could not perform isolated contractions of the three rectus segments, he could voluntarily generate different activation ratios between the three segments, which indicates a successful innervation of at least two of the compartments. Other neighboring muscles could have also influenced EMG signals, but the risk was minimized by placing the electrode centrally on the appropriate rectus segments. Muscle contractions in the transplant area were also confirmed by palpation. Furthermore, no signs of atrophy of the transplant could be observed since the time of transplantation. This further indicates a successful vascular supply and innervation of the transplant. After successful reinnervation, the intervention enabled the patient to generate reproducible and variable patterns of muscle contractions in the area of the transplant. With all residual muscles, including the transplant, seven classes could be distinguished with an error below 1%. The patient was successfully fitted with a pattern-recognition-based myoelectric prosthesis.

## 5. Conclusions

The RA muscle has been the working horse of reconstructive surgery for decades. The reduction of donor-site morbidity due to the minimal invasiveness of robotic surgery qualify the RA muscle as a game changer in amputation surgery due to its segmental architecture. An animal model using a pedicled RA demonstrated that TMR could generate a separate EMG signal in each muscle segment.

We have demonstrated, for the first time, that robotically harvested RA muscle can be transferred and reinnervated at the RL of a person a transradial amputation. The procedure resulted in additional independent EMG sites for myoelectric prosthesis control and improved soft tissue coverage of the distal part of the RL. Using the DaVinci system, harvesting the rectus muscle reduced the risk of donor-site morbidity. Twelve months after the intervention, we could confirm the success of the innervation process. The described procedure advances the currently existing peripheral nerve techniques (e.g., TMR) since the lower invasiveness of robotic surgery extends the application to more diverse amputation levels.

## Data Availability

The data presented in this study are available on request from the corresponding author.
